# Editorial: Differentiating brain cancers and glioblastoma through imaging methodologies

**DOI:** 10.3389/fonc.2023.1351874

**Published:** 2023-12-18

**Authors:** Ellen Ackerstaff, Pilar López-Larrubia

**Affiliations:** ^1^ Department of Cancer Systems Imaging, The University of Texas MD Anderson Cancer Center, Houston, TX, United States; ^2^ Instituto de Investigaciones Biomédicas Sols-Morreale, Consejor Superior de Investigaciones Científicas-Universidad Autónoma de Madrid (CSIC-UAM), Madrid, Spain

**Keywords:** neuro-oncology, multiparametric imaging, brain tumor differentiation, quantitative image analysis, radiomics, glioblastoma

In the field of neuro-oncology, the imperative to differentiate between various brain cancers, notably the formidable glioblastoma multiforme (GBM), is of paramount importance. As precision medicine becomes increasingly integrated into oncologic practices, advanced imaging methodologies emerge as indispensable tools for clinicians. These techniques provide a noninvasive insight into the intricate micro structures, molecular features, physiology, and metabolism of the brain, offering crucial information about the distinctive features of GBM and other brain cancers (1). The ability to discern subtle variations of tumor characteristics through noninvasive imaging not only facilitates accurate diagnosis but also establishes the foundation for personalized treatment strategies.

To treat GBM and other brain cancers as early as possible by successfully differentiating them from each other is essential to optimize their treatment, improving outcome (1–3). Emphasizing the fundamental role of imaging techniques in achieving this goal, the articles in this Research Topic focus on pretreatment imaging parameters and their analysis for the differentiation of brain cancer types.

As summarized in the Mini Review by Hooper and Ginat and others [e.g., (1, 4, 5)], no single imaging technique has been proven to be successful in this aim, whereas a combination of imaging modalities, such as e.g., ^18^F-FDG PET and multiparametric magnetic resonance (MR) imaging (MRI), improve the detection, staging and differentiation of brain tumors. Quantitative radiomics of multiparametric MRI with or without the inclusion of brain MR spectroscopy further the finer differentiation of brain tumors as well as glioma or GBM subtypes, with the goal of personalizing tumor treatment (1, Hooper and Ginat; Vallee et al.; Meier et al.). Specifically, as shown by Vallee et al., the analyses of multiparametric MR data using decision tree models show promise in differentiating brain metastasis, GBM, and primary central nervous system lymphomas (PCNSL), compared to other analyses approaches [e.g., (6)].

One main limitation of current quantitative radiomics is its lack of reproducible applicability across independent studies, mostly due to improper data portioning, overfitting, and a lack of standardization (Hooper and Ginat; Vallee et al.; Meier et al.). Combining training cohorts with independent testing cohorts is one way to establish the robustness of novel or new quantitative or semiquantitative imaging parameter models to differentiate brain tumor types, such as presented in the study by Han et al. with models separating PCNSL from GBM.

Linking radiomics features and underlying biology, by combining quantitative radiomics with radio–genomics for example, has the power to enhance brain tumor characterization and identify meaningful and reproducible radiomic signatures (Hooper and Ginat, 5). This has been of special interest for GBM patients, as the molecular subtype has been shown to be prognostic as well as influences treatment (2, 5). For example, isocitrate dehydrogenase (IDH)–mutant gliomas may benefit from temozolomide treatment and high–grade, IDH–1 mutant gliomas from including the non–contrast–enhancing, tumor–surrounding tissue in the surgical resection (5).

The Cancer Imaging Archive [TCIA, (7), last accessed on 12/05/2023] and the UK Biobank (last accessed on 12/05/2023) contain various imaging data as well as to the imaging related data, such as treatment details, genetic, biomarker and/or health–related outcome data. Thus, both data archives offer to be powerful tools in the development of quantitative or semiquantitative analysis models of multiparametric and multimodal *in vivo* and *ex vivo* imaging, combined with “radiohistogenomic interpretation”, for brain tumors as well as other cancers or diseases. Despite progress made, brain tumors remain difficult tumors to treat with low cure rates and several challenges for successful treatment to overcome (8), with noninvasive *in vivo* imaging playing a future role in linking radiomics to personalized treatment at time of diagnosis and in the monitoring of treatment response.

## Author contributions

EA: Writing – original draft, Writing – review & editing. PL–L: Writing – review & editing.
